# Avenanthramide-C Mitigates High-Fat Diet-Accelerated Alzheimer’s Pathologies via NOD1-Driven Neuroinflammation in 5×FAD Mice

**DOI:** 10.3390/nu17162679

**Published:** 2025-08-19

**Authors:** Ming Wang, Baoyuan Jin, Jia Xu, Chuang Wang

**Affiliations:** 1 Health Science Center, Ningbo University, Ningbo 315211, China; 2 Chonnam National University Medical School and Hospital, 42 Jebong-ro, Gwangju 61469, Republic of Korea; fyyjinbaoyuan@nbu.edu.cn; 3 The First Affiliated Hospital of Ningbo University, Ningbo 315211, China; 4 School of Medicine, Ningbo University, Ningbo 315211, China; xujia@nbu.edu.cn; 5 School of Basic Medical Science, Health Science Center, Ningbo University, Ningbo 315211, China

**Keywords:** avenanthramide-C, HFD-fed mice, neuroinflammation, synaptic plasticity, Alzheimer’s pathologies

## Abstract

**Background**: Obesity is clinically known to be associated with an increased risk and aggravated pathology of Alzheimer’s disease (AD). A high-fat diet (HFD), the major contributor to obesity, induces neuroinflammation and central insulin resistance, both of which are linked to synaptic dysfunction. Our previous studies demonstrated that avenanthramide-C (Avn-C), a natural oat-derived phenolic compound, exerts anti-inflammatory effects and alleviates synaptic dysfunction in conventional AD models. The present study aimed to elucidate the underlying mechanisms of Avn-C in obesity-accelerated AD. **Methods**: Two-month-old male 5×FAD mice were fed an HFD to induce obesity and then treated with Avn-C. Cognitive performance, synaptic function, and structure were assessed via behavioral tests, electrophysiological recordings, and Golgi–Cox staining, respectively. Cytokine levels were quantified using ELISA and Western blotting. To explore the underlying mechanism, the NOD1 agonist C12-iE-DAP was administered to evaluate its effect on Avn-C-mediated neuroprotection. **Results**: Avn-C reduced Aβ deposition, enhanced the expression of synapse proteins, and restored synaptic plasticity, thereby improving both spatial and recognition memory in obese 5×FAD mice. Furthermore, Avn-C reduced neuroinflammation by inhibiting the NOD1/RIP2/NF-κB signaling pathway. Co-treatment with C12-iE-DAP abolished the beneficial effects of Avn-C on neuroinflammation, Aβ pathology, and cognitive function. **Conclusions**: These results suggest that Avn-C mitigates obesity-exacerbated AD-like pathological features by suppressing NOD1/RIP2/NF-κB-mediated neuroinflammation and could be a new potential therapeutic strategy for obesity-associated AD.

## 1. Introduction

AD, the most common form of dementia, is an irreversible neurodegenerative disorder characterized by progressive cerebral amyloidosis, intraneuronal neurofibrillary tangles, and memory loss [1,2], associated with neuroinflammation and widespread neuronal loss. Recent studies suggest that Aβ and tau are not the primary drivers but rather the consequences of severe synaptic damage; however, persistent or excessive neuroinflammation has been considered a cause of disrupted synaptic plasticity and long-term memory deficits [3,4,5]. HFD is considered a major factor in obesity, and long-term HFD consumption leads to systemic inflammation and disrupted insulin signaling [6,7,8]. It has been proposed that HFD-induced neuroinflammation and central insulin resistance play direct roles in diminishing the clearance of extracellular Aβ, ultimately accelerating amyloid deposition in the brains of AD mice [9]. Numerous meta-analyses and longitudinal studies have reported that obese individuals consuming an HFD have poorer cognitive function and that individuals with middle-aged obesity or diabetes are at an increased risk of developing AD later in life [10,11,12].

Learning and memory rely on functional and structural changes in the hippocampus, such as synaptic plasticity and synaptic remodeling [13,14]. The hippocampus is one of the first brain regions affected in AD [15], exhibiting higher immune activity and susceptibility to inflammation than other brain regions [16]. Notably, the hippocampus appears particularly susceptible to damage induced by dietary factors [17,18,19]. Studies have demonstrated that as few as three days of HFD exposure can induce hippocampal neuroinflammation and cognitive impairment in aged rats [20]. The continued accumulation of inflammatory cytokines, such as IL-1β, IL-6, and TNF-α, impairs hippocampal LTP [21]. It has been reported that targeting NF-κB signaling confers a protective effect on diabetes and neurological diseases [22]. This suggests that HFD consumption, starting with its ingestion, negatively impacts hippocampal function throughout the lifespan.

Avenanthramides, the primary water-soluble phenol-amides uniquely present in oats, have been identified as key contributors to the antioxidant properties of whole grain oat consumption and their beneficial effects on human health [23,24]. It has also been shown that avenanthramides could inhibit postprandial glucose uptake [25]. A meta-analysis study revealed that regular oat intake reduces the incidence of T2DM [26]. Avn-C is one of the most abundant avenanthramides in oats, and its anti-inflammatory effects are widely recognized in various diseases in both humans and rodents [27,28,29,30,31]. The benefits of Avn-C in treating cognitive dysfunction and memory deterioration have been demonstrated in aging rats [32]. We previously reported that the oral administration of Avn-C effectively inhibited neuronal inflammation and reversed impaired synaptic plasticity in AD model mice [33]. However, the pharmacological potential of Avn-C in obesity-exacerbated AD pathological features remains unexplored.

In this study, we found that Avn-C significantly alleviated HFD-accelerated neuroinflammation, amyloid pathology, and cognitive deficits in 5×FAD mice. Notably, these effects were abolished via co-treatment with the NOD1 agonist C12-iE-DAP. Therefore, our findings demonstrate the significant potential of Avn-C, which may mitigate cognitive decline in HFD-fed AD mice by suppressing neuroinflammation through the inhibition of the NOD1/RIP2/NF-κB signaling pathway.

## 2. Materials and Methods

### 2.1. Animals

Transgenic mice, 5×FAD, were bred from progenitors obtained from Jackson Lab (Bar Harbor, ME, USA) (strain B6SJL-Tg, APPSwFlLon, PSEN1*M146L*L286V, 6799Vas/J, MMRRC). Animals were kept in individual cages with free access to food and water. The breeding room was controlled with a 12 h light/dark cycle and the temperature was maintained at 22–30 °C. All experiments were carried out according to the 1996 Guidance for Animal Experiments, and the procedures were approved by the Animal Care and Use Committee of Chonnam National University.

### 2.2. High-Fat Diet and Avn-C Treatment

The 5×FAD mice were randomly assigned to experimental groups, and blinding was applied during experimentation and data analysis. Mice in the HFD-fed groups received a high-fat diet for 16 weeks, from 2 to 6 months of age, covering the period from early amyloid pathology to cognitive decline and high amyloid deposition. The high-fat diet (#D12331, Research Diet Inc., New Brunswick, NJ, USA) consisted of a diet with fat and sucrose (protein: 17% kcal, fat: 58%, carbohydrate: 25%, energy density: 5.56 kcal/g). The HFD was supplemented with 4 g for each mouse per day, exceeding 20 kcal energy. Control mice were given a normal chow diet. The body weights (BWs) of all animals were tracked on a weekly basis.

**Figure 1:**
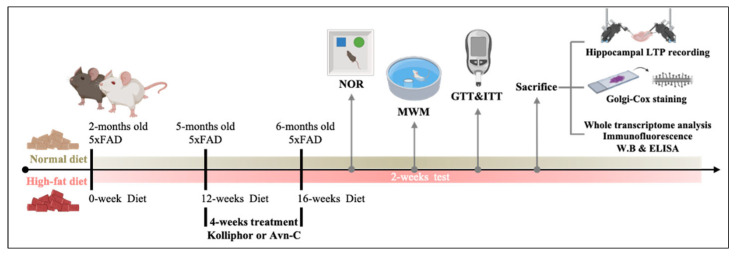
Graphical representation of experimental design. Two-month-old male 5×FAD mice were fed either a normal diet (control, non-HFD-treated 5×FAD group) or HFD. Animals were treated via intragastrical gavage, either with Kolliphor or with Avn-C, at a dose of 6 mg/kg for 4 weeks. Then, animals were subjected to a series of evaluations: NOR and MWM, GTT and ITT, electrophysiological recordings, Golgi–Cox staining, transcriptome analysis, immunofluorescence, Western blotting, and ELISA.

Our previous study showed a dose–response result when 6 mg/kg of Avn-C was orally administered before further assessment [33]. Avn-C was purchased from Sigma-Aldrich (#36465, Sigma-Aldrich, St. Louis, MO, USA). For treatment, a final concentration of 1 mg/mL Avn-C was prepared by dissolving it in 10% Kolliphor (Sigma-Aldrich, St. Louis, MO, USA). In the current study, at 5 months of age, Avn-C was administered by intragastrical gavage to HFD- and Avn-C-treated mice once a day (6 mg/kg) for 4 weeks. To control for potential circadian effects on drug pharmacodynamics, Avn-C was administered once daily at 15:00, ensuring temporal consistency throughout the experimental period. The experiments were conducted when the mice were 6 months of age. Figure 1 shows a graphical representation of the experimental design.

### 2.3. Intraperitoneal Glucose Tolerance Test (IPGTT)

The IPGTT is designed to determine the clearance of an intraperitoneally injected glucose load from the body. The 5×FAD mice were fasted for almost 16 h. Glucose (2 g/kg, dissolved in saline) was intraperitoneally injected. Blood glucose levels were measured at 0, 30, 60, and 120 min post-injection using a glucometer, with blood samples taken from the tip of the tail. The area under the curve (AUC) for the glucose concentration over the 0 to 120 min period (mg/dL * minutes) was then calculated.

### 2.4. Intraperitoneal Insulin Tolerance Test (IPITT)

Mice from each group were fasted for 4 h by taking away food early in the morning. They were given an intraperitoneal injection of insulin (0.75 U/kg, in saline), and blood samples were taken from the tip of the tail. Blood glucose levels were measured by a glucometer at 0, 15, 30, 60, and 120 min post-injection. Between each of these time points, the mouse was returned to its cage and monitored. The area under the glucose concentration–time curve (AUC glucose 0–120 min, mg/dL * minutes) was then calculated.

### 2.5. ELISA for Insulin and Cytokines

Hippocampal tissue was homogenized in ice-cold PBS containing protease inhibitors, centrifuged at 13,000 rpm for 20 min at 4 °C, and the supernatants collected for analysis. Hippocampal insulin was measured with an insulin ELISA kit (Shibayagi, Gumma, Japan). First, 10 μL each of the standard and sample was added to appropriate wells. Next, 75 μL of enzyme conjugate was added to each well, and they were incubated for 2 h by shaking at 700 rpm at room temperature (RT). Microplates were washed 5 times with wash buffer. Then, 100 μL of substrate solution was added to each well, and they were incubated for 15 min at RT. The reaction was stopped by adding 100 μL/well of stop solution, and the absorbance was detected at 450 nm using a microplate reader.

The concentrations of TNF-α, IL-1β, IL-6, and IL-10 in hippocampal tissue were measured using ELISA kits (Mouse TNF-α, IL-1β, IL-6, and IL-10 Ready-SET-Go, eBioscience, San Diego, CA, USA), following the manufacturer’s instructions. Briefly, ELISA plates were coated with 100 μL/well of capture antibody in coating buffer and incubated overnight at RT. Microplates were washed 3 times with wash buffer. Then, 300 μL/well of reagent diluent was added for blocking at RT for 1 h. Next, 100 μL each of the standard and sample was added to appropriate wells, and they were incubated at RT for 2 h. After washing 5 times, 100 μL of detection antibody was added, and they were incubated for 2 h at RT. The wells were washed 5 times, followed by the addition of 100 μL/well of streptavidin–HRP and incubation for 20 min at RT. Then, 100 μL of substrate solution was added to each well. After 15 min, the reaction was stopped with 50 μL of stop solution, and the absorbance was measured at 450 nm using a microplate reader.

### 2.6. Novel Objective Recognition Test

The NOR test serves as a test of short-term object recognition memory in experimental animals. The procedure consisted of adaptation, familiarization, and testing phases. Animals were placed in the testing apparatus to acclimate to the new environment 24 h prior to the experiment. On the first day, each mouse was allowed to freely habituate to an empty apparatus measuring 40 × 40 × 40 (cm) for 10 min individually. On the second day, two identical objects (3 cm in diameter) were placed in the apparatus, positioned 10 cm away from each wall. Each mouse was introduced to the apparatus facing the wall furthest from the objects and allowed to explore for 5 min, and the entire session was video-recorded via a system that automatically captured and collected data. To eliminate olfactory cues, the apparatus was cleaned with 70% alcohol between sessions. In the testing stage on the third day, one of the blocks was replaced with a new object of a different shape but identical color and texture, while retaining its original location. Each mouse was again introduced to the apparatus, facing the wall furthest from the objects, and allowed a 5 min exploration period. Exploration was defined as the mouse directing its nose towards the object, with a nose-to-object distance of less than 2 cm, excluding time spent sitting on or resting near the object. The final data recorded included the time that each mouse spent exploring the novel object and the familiar object. Upon completion of the experiment, the discrimination index (*DI*) was calculated to quantify recognition memory performance in 5×FAD mice:

$$DI=\frac{{Time}_{novel}-{Time}_{familiar}}{{Time}_{novel}+{Time}_{familiar}}$$

### 2.7. Morris Water Maze Test

Spatial memory was tested using the Morris water maze test. This assessment comprised a 5-day acquisition period, after which a probe test was performed 24 h later. The 5×FAD mice were tested in a circular pool filled with water mixed with titanium oxide, and the temperature was maintained at 22–23 °C. The pool was partitioned into four quadrants of equal size. One quadrant was chosen to mount a submerged object (2 cm beneath the water surface). In the acquisition sessions, mice were introduced into the pool, facing the wall, from the four quadrants of the pool. The recording time was 1 min. If the animal found the platform and remained on it for 3 s, it was considered to have located the platform, and the recording was automatically stopped. The time taken to escape the system was recorded. If the mouse did not locate the platform within the designated time, it was gently guided to the platform and remained on the platform for 30 s before being removed. After each trial, the mice were dried using a hairdryer and returned to their respective cages. After 24 h, a spatial probe trial was performed in the absence of the platform. The quadrant opposite the platform’s location was selected as the entry point, and the trial was recorded for 1 min. The percentage of time that the mouse spent in the platform quadrant relative to the total time spent in all quadrants was calculated.

### 2.8. Hippocampal Slice Preparation

The 5×FAD mice were sacrificed by cervical dislocation and then decapitated. Brains were quickly extracted and immersed in ice-chilled artificial cerebrospinal fluid (aCSF) composed of 124 mM NaCl, 3 mM KCl, 26 mM NaHCO_3_, 1.25 mM NaH_2_PO_4_, 2 mM CaCl_2_, 1 mM MgSO_4_, and 10 mM glucose. The hippocampus was transversely sliced into sections of 400 μm thickness using a McIlwain tissue chopper (Mickle Laboratory Engineering Co., Ltd., Guildford, Surrey, UK). The sections were then stabilized for 1 h in oxygenated aCSF (95% O_2_/5% CO_2_ mixture) at RT.

### 2.9. Electrophysiological Recordings

Hippocampal slices were placed in a recording chamber continuously perfused with oxygenated aCSF (28–29 °C). Field excitatory postsynaptic potentials (fEPSPs) were evoked by placing a bipolar stimulating electrode. The field EPSP was assessed with a glass microelectrode prepared with a micropipette puller (P-1000; Sutter Instruments, Novato, CA, USA) and filled with 3 M NaCl, yielding tip resistance of 3–5 MΩ. After establishing a stable baseline for 30 min, LTP was induced using two tetanic trains (100 Hz for 1 s with a 30 s interval). The EPSPs were recorded for 1 h. Signals were acquired through an NI USB-6251 module (National Instruments, Austin, TX, USA), amplified with an Axopatch 700B (Axon Instruments, San Jose, CA, USA), and analyzed using the WinLTP software 2.32 (http://www.winltp.com) (accessed on 1 July 2021).

### 2.10. Golgi–Cox Staining and Spine Density Analysis

Golgi–Cox staining was performed using a commercial rapid kit (FD Rapid Golgi Stain Kit, FD NeuroTechnologies, Inc., Columbia, MD, USA), following the manufacturer’s instructions. In brief, brains were dissected and fixed in 4% paraformaldehyde for 24 h. The tissue was cut into ~2 mm coronal blocks, rinsed in 1X PBS for 5 min, and then immersed in an impregnation mixture (equal volumes of solutions A and B, prepared one day in advance). Samples were kept at room temperature in darkness for two weeks, followed by transfer to solution C at 4 °C for an additional three days. Subsequently, hippocampal sections of approximately 100 μm thickness were generated using a McIlwain tissue chopper and mounted on gelatin-coated glass slides with a drop of solution C. After natural air-drying overnight at RT, the sections were treated with developer solution (solution D mixed with double-distilled water at 1:1:2) for 10 min, dehydrated through graded ethanol (50%, 75%, 95%, 100%), and sealed under coverslips. Images of dendritic arbors in the CA1 hippocampal region were acquired using the CaseViewer 2.3.0 system (3DHISTECH, Budapest, Hungary). For each group, 12 pyramidal neurons (four per CA1 subregion) were selected, ensuring clear separation from adjacent cells. Dendritic segments (70–100 μm in length) from both basal and apical regions were analyzed. The total number and length of dendritic spines were measured using ImageJ 1.54i (NIH, Bethesda, MD, USA), and the spine density was calculated as the number of spines divided by the dendritic segment length.

### 2.11. Western Blots of Hippocampus

The hippocampus was treated with cold RIPA buffer with a protease and phosphatase inhibitor cocktail (#1861281, Thermo Scientific, Waltham, MA, USA). The protein concentration was determined using the BCA method. A total of 30–40 μg of protein was separated on 8–10% SDS–polyacrylamide gels and electrotransferred onto PVDF membranes (Millipore, Bedford, MA, USA). The blots were incubated for 16 h at 4 °C with primary antibodies (at 1:1000 dilutions) specific for p-GSK3β (Ser9) (#5558, D85E12, Cell Signaling, Danvers, MA, USA), GSK-3β (#5676, D75D3, Cell Signaling), p-Akt (Ser473) (#4060, D9E, Cell Signaling), Akt (#9272, C67E7, Cell Signaling), p-IRS-1 (Tyr612) (#44-816G, polyclonal antibody, Invitrogen, California, CA, USA), IRS-1 (#PA1-1057, polyclonal antibody, Invitrogen), Shank3 (#sc-377088, C-4, Santa Cruz, Dallas, TX, USA), PSD95 (#3450, D27E11, Cell Signaling), GluA1 (#13185, 13185, Cell Signaling), GluN1 (#5704, D65B7, Cell Signaling), GluN2B (#4212, D15B3, Cell Signaling), TNF-α (#11948, D2D4, Cell Signaling), IL-1β (#12507, D3H1Z, Cell Signaling), IL-6 (#12153, D3K2N, Cell Signaling), IL-10 (#12163, D13A11, Cell Signaling), NOD1 (#3545, polyclonal antibody, Cell Signaling), RIP2 (#4142, D10B11, Cell Signaling), p-p65 (Ser536) (#3033, 93H1, Cell Signaling), NF-κB (p65) (#8242, D14E12, Cell Signaling), or β-actin (#ab8227, polyclonal antibody, Abcam, Cambridge, MA, USA). The immunoblots were then incubated with specific secondary antibodies (Abcam) for 2 h at RT, and the bands were detected with an enhanced chemiluminescence device (Millipore, Billerica, MA, USA). Protein expression was measured using the Fusion Solo imaging system and normalized to β-actin and total protein levels.

### 2.12. Immunofluorescence

Brain slices (20 μm) were placed onto collagen-coated glass slides (Thermo Scientific, Waltham, MA, USA). The tissues were fixed in acetone for 10 min, rinsed with Tris-buffered saline, and subsequently immersed in methanol for 5 min. To reduce nonspecific binding, the sections were preincubated in 5% bovine serum albumin (Sigma-Aldrich) for 1 h. This was followed by overnight incubation at 4 °C with primary antibodies diluted in PBS (1:500 dilutions): Iba1 (#019-19741, Wako, Osaka, Japan), GFAP (#Z0334, Dako, Santa Clara, CA, USA), and primary antiserum to NeuN (#MAB377, Millipore, Burlington, MA, USA) (1:1000). The samples were washed three times for 5 min each in PBS containing 0.1% Tween 20 and then incubated for 2 h at room temperature in the dark with secondary antibodies: Alexa Fluor 488-conjugated goat anti-rabbit IgG and Alexa Fluor 594-conjugated goat anti-mouse IgG (1:500; Invitrogen). Cell nuclei were counterstained with DAPI (1 μg/mL; Sigma-Aldrich), and the slides were imaged using a confocal microscope (Carl Zeiss, Oberkochen, Germany).

### 2.13. Microarray Analysis and Differential Gene Expression Analysis

Total RNA was extracted from hippocampi and purified using the RNeasy Plus Mini Kit (#74134, Qiagen, Hilden, Germany), according to the manufacturer’s instructions. Then, the mRNA samples were reversed-transcribed and labeled with a fluorescent dye and hybridized on the array. Transcriptome Analysis Console (TAC) combines the CEL file analysis and QC features of Expression Console and the statistical analysis of TAC. The results were summarized and normalized using Affymetrix Power Tools (APT). These data were exported for DEG analysis. The statistical significance of the expression data was determined using an independent *t*-test and fold change (FC), in which the null hypothesis was that no difference would exist among the groups. The false discovery rate (FDR) was controlled via the *p*-value using the Benjamini–Hochberg algorithm. Genes with |FC| > 1.2 and *p*-value < 0.05 were classified as significantly differentially regulated. GO enrichment analysis was performed using R version 4.4.0. For visualization, volcano plots, bubble charts, and Circos plots were constructed using ggplot2, R version 4.4.0.

### 2.14. C12-iE-DAP Administration

C12-iE-DAP, a NOD1 agonist (tlrl-c12dap), was obtained from InvivoGen. C12-iE-DAP was dissolved in dimethyl sulfoxide (DMSO) to final concentrations of 0.5 mM, 1 mM, and 2 mM and was subsequently administered to obese 5×FAD mice via intracerebroventricular (i.c.v.) injection (coordinates: 1.5 mm posterior, 1.0 mm lateral, and 3.2 mm ventral relative to bregma) once every three days over a period of four weeks.

### 2.15. Data Analysis

Statistical analyses were conducted using SPSS 21.0 (IBM Corp., Armonk, NY, USA). Data were expressed as the mean ± standard error of the mean (S.E.M.), and the Shapiro–Wilk test was used to assess the normality of the data distribution. For comparisons between two groups, an unpaired Student’s *t*-test was used. For comparisons among three or more groups, a one-way analysis of variance (ANOVA) was conducted, followed by Tukey’s post hoc test. Significant differences were considered at *p* < 0.05; otherwise, they were considered not significant (ns). Effect sizes for ANOVA are presented as η^2^ values.

## 3. Results

### 3.1. Avn-C Does Not Reverse Body Weight Gain or Glucose Tolerance but Does Alleviate Insulin Resistance in Obese 5×FAD Mouse Hippocampi

**Figure 2:**
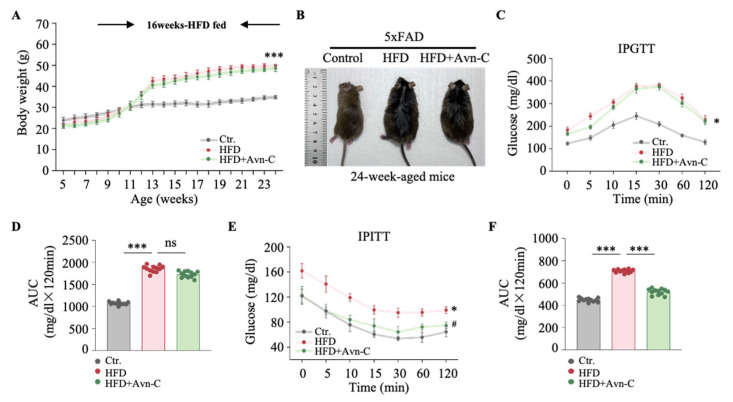
Avn-C did not change body weight and glucose metabolism in obese 5×FAD mice. (**A**) Body weight change during the 16-week intervention (n = 12 per group from twelve animals). One-way ANOVA: F (2, 33) = 20.7, *p* = 0.0002. (**B**) Pictures of 24-week-old mice from each group. (**C**) Blood glucose levels were monitored over time after the intraperitoneal glucose tolerance test (IPGTT) in 5×FAD mice from each group (n = 12 per group from twelve animals). One-way ANOVA: F (2, 33) = 19.6, *p* = 0.0003. (**D**) Calculation of area under the curve after IPGTT (n = 12 per group from twelve animals). One-way ANOVA: F (2, 33) = 24.1, *p* < 0.0001. (**E**) Blood glucose levels were monitored over time after the intraperitoneal insulin tolerance test (IPITT) in 5×FAD mice from each group (n = 12 per group from twelve animals). One-way ANOVA: F (2, 33) = 15.3, *p* < 0.0001. (**F**) Calculation of area under the curve after IPITT (n = 12 per group from twelve animals). One-way ANOVA: F (2, 33) = 19.4, *p* < 0.0001. Data are expressed as means ± S.E.M., and statistical analysis was performed by Tukey’s post hoc test, **^#^** *p* < 0.05, * *p* < 0.05, *** *p* < 0.001.

To evaluate the effect of Avn-C on HFD-induced weight gain, 5×FAD mice were fed an HFD for 16 weeks, with Avn-C for 4 weeks. The HFD induced significant weight gain in 5×FAD mice (HFD vs. the control, *p* < 0.001) (Figure 2A), and Avn-C treatment did not significantly reverse the weight gain induced by the HFD (HFD + Avn-C vs. HFD, *p* > 0.05) (Figure 2A). Pictures of 24-week-old mice from each group are presented in Figure 2B. To determine the effects of Avn-C on glucose metabolism, the IPGTT was performed. Obese 5×FAD mice exhibited glucose tolerance compared with the controls (*p* < 0.05) (Figure 2C). Avn-C treatment did not improve glucose metabolism in HFD-fed mice (HFD + Avn-C vs. HFD, *p* > 0.05) (Figure 2C), and the same observation was made in the quantification of the area under the curve (AUC) (HFD + Avn-C vs. HFD, *p* > 0.05) (Figure 2D). To further explore the effects of Avn-C on insulin sensitivity, an IPITT was conducted. We found that obese 5×FAD mice displayed significant insulin resistance compared with the controls (*p* < 0.05) (Figure 2E). However, Avn-C treatment markedly improved insulin sensitivity, with glucose levels decreasing more efficiently in Avn-C-treated mice than in untreated HFD-fed mice (*p* < 0.05) (Figure 2E). The calculated AUC values (HFD vs. the control, *p* < 0.001; HFD + Avn-C vs. HFD, *p* < 0.001) (Figure 2F) further confirmed Avn-C’s effect in mitigating insulin resistance in obese 5×FAD mice.

**Figure 3:**
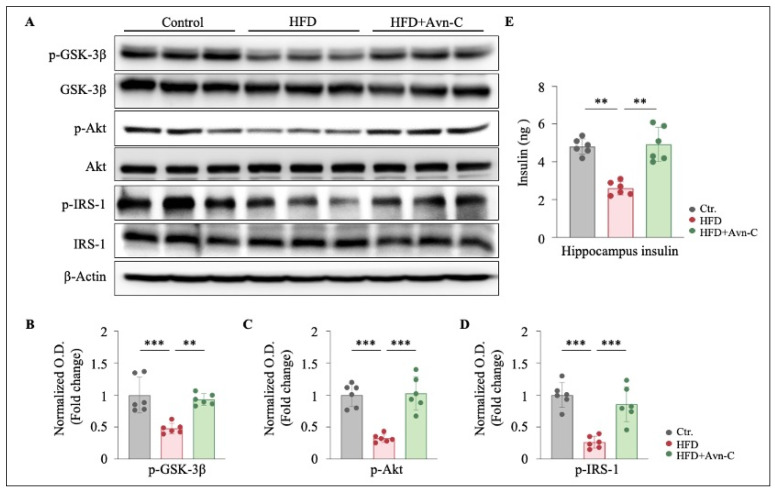
Avn-C activates GSK-3β/Akt signaling and rescues insulin resistance in obese 5×FAD. (**A**) Representative immunoblots showing the protein levels of p-GSK-3β, GSK-3β, p-Akt, Akt, p-IRS-1, and IRS-1 in the hippocampus. (**B**–**D**) Densitometry analysis and quantification of Western blots (n = 6 per group from six animals). One-way ANOVA: F (2, 15) = 15.3, *p* = 0.0002, η^2^ = 0.67; F (2, 15) = 27.2, *p* < 0.0001, η^2^ = 0.78; F (2, 15) = 22.5, *p* < 0.0001, η^2^ = 0.75. (**E**) ELISA result showing the hippocampal insulin levels in each group (n = 6 per group from six animals). One-way ANOVA: F (2, 15) = 27.6, *p* < 0.0001, η^2^ = 0.79. Data are expressed as means ± S.E.M., and statistical analysis was performed via Tukey’s post hoc test, ** *p* < 0.01, *** *p* < 0.001.

To investigate the effects of Avn-C in the HFD-induced disruption of hippocampal insulin signaling, the phosphorylation levels of key signaling molecules were analyzed via Western blotting. The results show that obese 5×FAD mice displayed significant reductions in phospho-glycogen synthase kinase-3β (p-GSK-3β), phospho-protein kinase B (p-Akt), and phospho-insulin receptor substrate-1 (p-IRS-1) protein levels in the hippocampus (p-GSK-3β, *p* < 0.001 vs. the control; p-Akt, *p* < 0.001 vs. the control; and p-IRS-1, *p* < 0.001 vs. the control) (Figure 3A–D). These changes suggest impaired insulin signaling in the hippocampus in HFD-fed mice. Remarkably, Avn-C treatment significantly reversed the reductions in p-GSK-3β, p-Akt, and p-IRS-1 in obese 5×FAD mouse hippocampi (p-GSK-3β, *p* < 0.01 vs. the control; p-Akt, *p* < 0.001 vs. the control; and p-IRS-1, *p* < 0.001 vs. the control) (Figure 3A–D), indicating improved insulin signaling as a result of Avn-C treatment. Additionally, to evaluate the beneficial effect of Avn-C on HFD-induced hippocampal insulin resistance, the hippocampal insulin levels were quantified using an ELISA assay. The amount of hippocampal insulin was significantly reduced in obese 5×FAD mice but remarkably increased after Avn-C treatment (HFD vs. the control, *p* < 0.01; HFD + Avn-C vs. HFD, *p* < 0.01) (Figure 3E). These findings suggest that Avn-C effectively restores insulin sensitivity in obese 5×FAD mice.

### 3.2. Avn-C Reduces Recognition and Spatial Memory Impairments in Obese 5×FAD Mice

**Figure 4:**
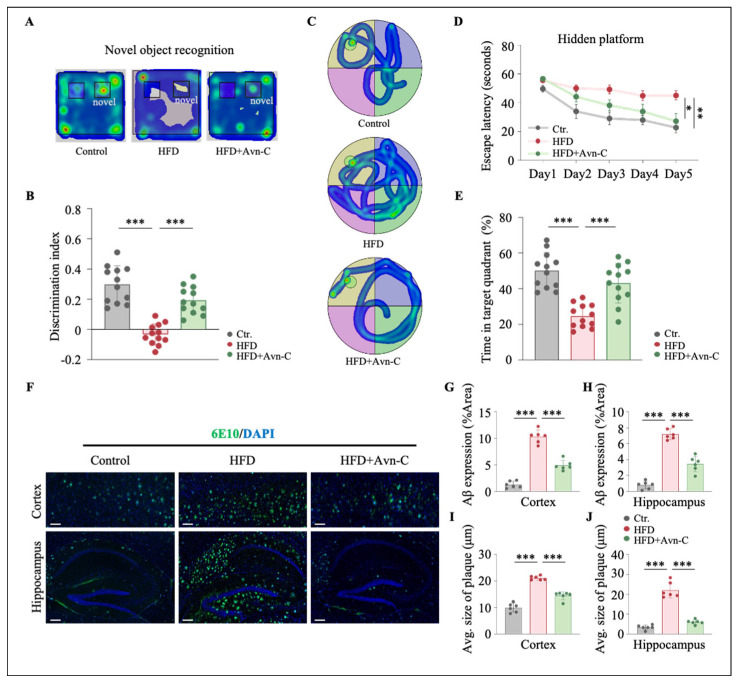
Avn-C protects 5×FAD mice from HFD-exacerbated memory deficit and Aβ deposition. (**A**) Heatmap plots show the average cumulative time that 5×FAD mice spent in different parts in the NOR session. Red indicates longer times. Blue indicates shorter times. (**B**) Discrimination index expressed in seconds (n = 12 per group from twelve animals). One-way ANOVA: F (2, 33) = 36.5, *p* < 0.0001, η^2^ = 0.69. (**C**) Heatmap plots show the escape strategy during the hidden platform task. Red indicates longer times. Blue indicates shorter times. (**D**) Escape latency and (**E**) time in the target quadrant (%) (n = 12 per group from twelve animals). One-way ANOVA: F (2, 33) = 16.0, *p* < 0.0001, η^2^ = 0.49; F (2, 33) = 23.3, *p* < 0.0001, η^2^ = 0.59. (**F**) Representative images of Aβ plaque immunofluorescence staining in the cortex and hippocampus from each group. Scale bar = 200 μm. (**G**,**H**) Quantification of Aβ deposition (% Area) based on immunofluorescence staining in the cortex and hippocampus (n = 6 per group from six animals). One-way ANOVA: F (2, 15) = 129.3, *p* < 0.0001, η^2^ = 0.95; F (2, 15) = 112.5, *p* < 0.0001, η^2^ = 0.94. (**I**,**J**) Average size of Aβ plaques (μm) was calculated based on immunofluorescence staining in the cortex and hippocampus (n = 6 per group from six animals). One-way ANOVA: F (2, 15) = 98.4, *p* < 0.0001, η^2^ = 0.93; F (2, 15) = 99.3, *p* < 0.0001, η^2^ = 0.93. Data are expressed as means ± S.E.M., and statistical analysis was performed via Tukey’s post hoc test, * *p* < 0.05, ** *p* < 0.01, *** *p* < 0.001.

Given that Avn-C attenuated insulin resistance and Aβ deposition in obese 5×FAD mice, we next investigated whether Avn-C treatment could also impact recognition and spatial learning and memory. To address this question, the novel objective recognition test was conducted at 6 months of age; the obese 5×FAD mice exhibited a lower discrimination index, indicating that their recognition memory was impaired (HFD vs. the control, *p* < 0.001) (Figure 4A,B). Avn-C treatment significantly enhanced the discrimination index in obese 5×FAD mice (HFD + Avn-C vs. HFD, *p* < 0.001) (Figure 4A,B). In addition, the Morris water maze test was performed; the 5×FAD mice were trained to find a hidden platform for five consecutive days and then subjected to a probe test with a platform. Figure 4C presents representative heatmaps of the escape strategy during the hidden platform task. During training, obese 5×FAD mice exhibited significantly longer escape latency to the platform in comparison with the 5×FAD controls (*p* < 0.01) (Figure 4D), indicating that HFD administration significantly accelerated memory impairment in 5×FAD mice. The capacity of Avn-C to ameliorate spatial memory decline in 5×FAD mice was also evident, where the Avn-C treatment significantly shortened the escape latency in obese 5×FAD mice compared with untreated obese mice (*p* < 0.05) (Figure 4D). During the probe trial assay at 24 h after the last training step, obese 5×FAD mice exhibited a weaker preference for the target quadrant among all quadrants (HFD vs. the control, *p* < 0.001) (Figure 4E). However, the time spent in the target quadrant was remarkably elevated in Avn-C-treated obese 5×FAD mice (HFD + Avn-C vs. HFD, *p* < 0.001) (Figure 4E). These results illustrate that Avn-C treatment significantly improved recognition memory and spatial memory in obese 5×FAD mice.

### 3.3. Avn-C Treatment Reduces Aβ Aggravation in Obese 5×FAD Mice

We next assessed whether Avn-C attenuated Aβ deposition in obese 5×FAD mice by performing immunofluorescence staining using an anti-Aβ (6E10) antibody on brain sections from 5×FAD mice. Moreover, either the Aβ expression or the average size of the Aβ plaque was quantified. As shown in Figure 4F, obese 5×FAD mice exhibited significant elevations in Aβ expression in the cortex and hippocampal regions compared with the 5×FAD controls (*p* < 0.001) (Figure 4G,H), as well as an HFD-induced increase in the size of Aβ plaques in both the cortex and hippocampal regions compared with the 5×FAD controls (*p* < 0.001) (Figure 4I,J), indicating the exacerbation of amyloid pathology as a result of the HFD in 5×FAD mouse brains. Importantly, Avn-C treatment significantly reduced both cortex and hippocampal Aβ expression and the size of Aβ plaques in obese 5×FAD mice (HFD + Avn-C vs. HFD, *p* < 0.001) (Figure 4G–J). Therefore, our results demonstrate that Avn-C mitigates the HFD-induced exacerbation of Aβ deposition in the 5×FAD mouse brain.

### 3.4. Avn-C Improves Synaptic Plasticity and Dendritic Spine Density in Obese 5×FAD Mice

**Figure 5:**
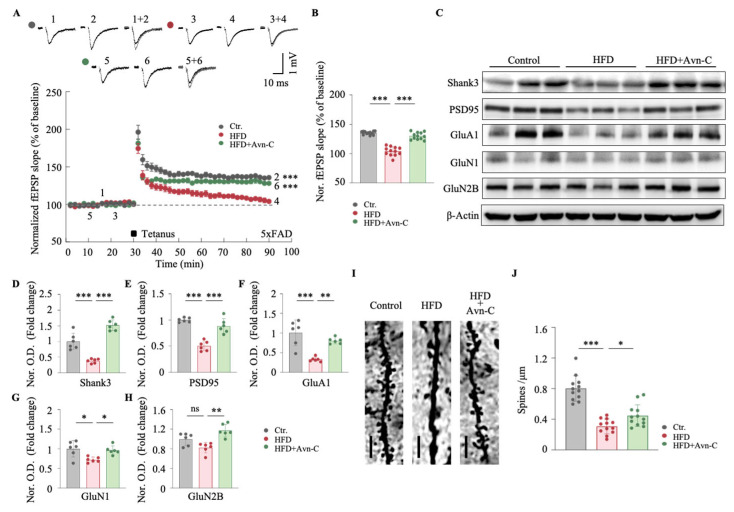
Avn-C facilitated synaptic plasticity and promoted the expression of synapse-related proteins and dendritic spines. (**A**) Facilitation of LTP induction (tetanus: 2-times tetanic stimulation at 100 Hz, 100 pulses, 30 s) in the CA1 region in 5×FAD mice hippocampal slices from each group. Control 5×FAD mice (Ctr, gray circles), obese 5×FAD mice (HFD, red circles), and obese 5×FAD mice treated with Avn-C (HFD + Avn-C, green circles); n = 12 per group from twelve animals. One-way ANOVA: F (2, 33) = 67.8, *p* < 0.0001, η^2^ = 0.80. (**B**) Bar graph of normalized fEPSP slopes from each group. (**C**) Representative immunoblots showing the protein levels of Shank3, PSD95, GluA1, GluN1, and GluN2B in the hippocampus. (**D**–**H**) Densitometry analysis and quantification of Western blots (n = 6 per group from six animals). One-way ANOVA: F (2, 15) = 56.2, *p* < 0.0001, η^2^ = 0.88; F (2, 15) = 39.0, *p* < 0.0001, η^2^ = 0.84; F (2, 15) = 18.7, *p* < 0.0001, η^2^ = 0.71; F (2, 15) = 6.7, *p* < 0.0001, η^2^ = 0.47; F (2, 15) = 12.8, *p* < 0.0001, η^2^ = 0.63. (**I**) Golgi–Cox staining was used to detect the morphology of CA1 pyramidal neurons. Representative dendritic segments are reported for each group (100× magnification, scale bar = 5 μm). (**J**) The plots represent the average density of spines calculated for each group. n = 12 dendritic segments from six animals per group. One-way ANOVA: F (2, 33) = 40.7, *p* < 0.0001, η^2^ = 0.71. Data are expressed as means ± S.E.M., and statistical analysis was performed via Tukey’s post hoc test, * *p* < 0.05, ** *p* < 0.01, *** *p* < 0.001.

The major pathological features of AD also include hippocampal synaptic structural and functional impairments [34]. To evaluate the effects of Avn-C on synaptic plasticity, fEPSPs were recorded at the hippocampal CA1 region, and LTP was induced using a high-frequency stimulation (HFS; two trains of 100 Hz, 100 pulses) protocol. For data acquisition, LTP was recorded for 60 min post-HFS. The results show that obese 5×FAD mice exhibited significantly impaired LTP compared with the 5×FAD controls, as evidenced by reduced fEPSP slopes following the HFS protocol (HFD vs. the control, *p* < 0.001) (Figure 5A,B). Notably, Avn-C treatment restored LTP in obese 5×FAD mice, with the fEPSP slopes significantly increased compared with untreated HFD-fed mice (HFD + Avn-C vs. HFD, *p* < 0.001) (Figure 5A,B), approaching the levels of the 5×FAD controls.

To further investigate the molecular mechanisms underlying the synaptic protective effects of Avn-C, the hippocampal levels of synaptic-related proteins were analyzed via Western blotting. The representative images and quantitative results show that obese 5×FAD mice displayed significantly decreased expression of Shank3, postsynaptic density protein 95 (PSD95), and GluA1 compared with the 5×FAD controls (Shank3, *p* < 0.001 vs. the control; PSD95, *p* < 0.001 vs. the control; and GluA1, *p* < 0.001 vs. the control) (Figure 5C–G; GluN1, *p* < 0.05 vs. the control), but no significant reduction in GluN2B protein levels was observed in the hippocampi of obese 5×FAD mice (GluN2B, *p* > 0.05 vs. the control) (Figure 5C,H). However, Avn-C treatment significantly restored the expression of these synaptic proteins, including Shank3, PSD95, GluA1, GluN1, and GluN2B, in obese 5×FAD mice (Shank3, *p* < 0.001 vs. HFD; PSD95, *p* < 0.001 vs. HFD; GluA1, *p* < 0.01 vs. HFD; GluN1, *p* < 0.05 vs. HFD; and GluN2B, *p* < 0.01 vs. HFD) (Figure 5C,D–H).

To assess whether Avn-C mitigates HFD-induced structural synaptic impairments, the dendritic spine density in the hippocampal CA1 region was analyzed. Representative images and the quantification of Golgi-stained dendrites revealed a significant reduction in spine density in obese 5×FAD mice (HFD vs. the control, *p* < 0.001) (Figure 5I,J). Avn-C treatment significantly increased the spine density in obese 5×FAD mice (HFD + Avn-C vs. HFD, *p* < 0.05) (Figure 5I,J). These results illustrate that Avn-C treatment alleviates HFD-induced synaptic plasticity deficits, rescues the expression of synaptic-related proteins, and improves the density of dendritic spines in HFD-fed 5×FAD mice.

### 3.5. Avn-C Inhibits Neuronal Inflammation Induced by HFD

**Figure 6:**
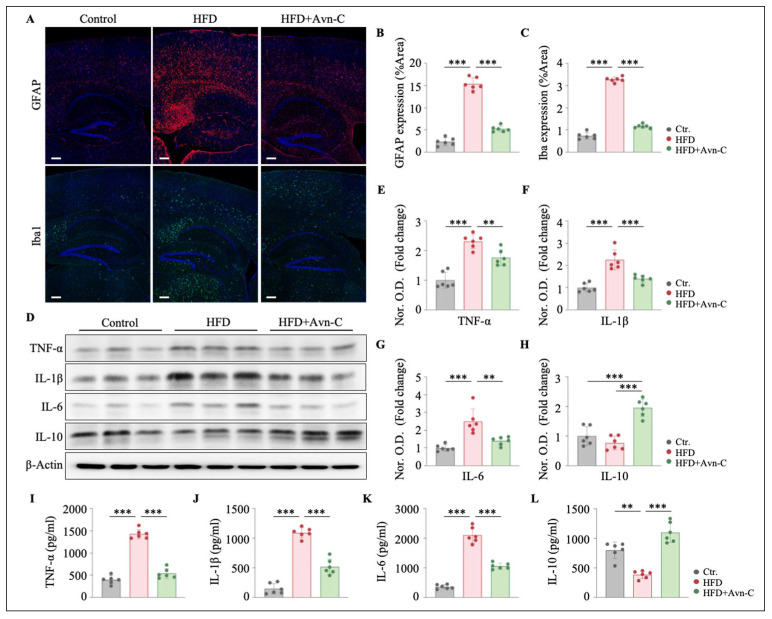
Avn-C reduced the neuroinflammatory response. (**A**) Representative images of GFAP and Iba1 immunofluorescence staining in brain slices from each group. Scale bar = 200 μm. (**B**,**C**) Quantification of GFAP and Iba1 immunofluorescence is presented as the mean fluorescence intensity (% Area) (n = 6 per group from six animals). One-way ANOVA: F (2, 15) = 266.8, *p* < 0.0001, η^2^ = 0.97; F (2, 15) = 241.4, *p* < 0.0001, η^2^ = 0.97. (**D**) Representative immunoblots showing the protein levels of TNF-α, IL-1β, IL-6, and IL-10 in the hippocampus. (**E**–**H**) Densitometry analysis and quantification of Western blots. One-way ANOVA: F (2, 15) = 38.1, *p* < 0.0001, η^2^ = 0.84; F (2, 15) = 27.7, *p* < 0.0001, η^2^ = 0.79; F (2, 15) = 18.3, *p* < 0.0001, η^2^ = 0.71; F (2, 15) = 29.6, *p* < 0.0001, η^2^ = 0.80. (**I**–**L**) ELISA results show the levels of TNF-α, IL-1β, IL-6, and IL-10 in the hippocampus (n = 6 per group from six animals). One-way ANOVA: F (2, 15) = 173.3, *p* < 0.0001, η^2^ = 0.96; F (2, 15) = 125.3, *p* < 0.0001, η^2^ = 0.94; F (2, 15) = 159.7, *p* < 0.0001, η^2^ = 0.96; F (2, 15) = 43.5, *p* < 0.0001, η^2^ = 0.85. Data are expressed as means ± S.E.M., and statistical analysis was performed via Tukey’s post hoc test, ** *p* < 0.01, *** *p* < 0.001.

The inflammatory environment triggered by Aβ accumulation shifts glia toward a pro-inflammatory phenotype; consequently, the activation of microglia and astrocytes accelerates the progression of AD [35]. Next, we examined whether Avn-C mitigated HFD-induced neuroinflammation. Immunofluorescence staining for GFAP (astrocyte marker) and Iba1 (microglial marker) was performed (Figure 6A). We found the robust activation of astrocytes and microglia in the brain sections of obese 5×FAD mice, as indicated by a significant increase in the GFAP fluorescence intensity (HFD vs. the control, *p* < 0.001) (Figure 6B) and Iba1 fluorescence intensity in obese 5×FAD mice (HFD vs. the control, *p* < 0.001) (Figure 6C). In obese 5×FAD mice treated with Avn-C, we observed a significant reduction in the expression levels of both GFAP and Iba1 (GFAP, *p* < 0.001 vs. HFD; Iba1, *p* < 0.001 vs. HFD) (Figure 6B,C).

To further corroborate the anti-inflammatory effects of Avn-C, the expression of pro-inflammatory cytokines was evaluated via Western blotting. The increased levels of tumor necrosis factor-alpha (TNF-α), interleukin-1 beta (IL-1β), and IL-6 in the hippocampus in response to the HFD (TNF-α, *p* < 0.001 vs. the control; IL-1β, *p* < 0.001 vs. the control; and IL-6, *p* < 0.001 vs. the control) (Figure 6D–G) were significantly reduced in response to Avn-C (TNF-α, *p* < 0.01 vs. HFD; IL-1β, *p* < 0.001 vs. HFD; and IL-6, *p* < 0.01 vs. HFD) (Figure 6D–G). We also observed an elevation in IL-10 expression following Avn-C treatment (*p* < 0.01 vs. the control; *p* < 0.001 vs. HFD) (Figure 6H). In addition, the ELISA results suggested that Avn-C might be associated with regulating the balance between pro-inflammatory and anti-inflammatory cytokines in the hippocampi of obese 5×FAD mice (TNF-α, *p* < 0.001 vs. HFD; IL-1β, *p* < 0.001 vs. HFD; IL-6, *p* < 0.001 vs. HFD; and IL-10, *p* < 0.001 vs. HFD) (Figure 6I–L). Collectively, these results indicate that central immune homeostasis is disrupted in HFD-fed 5×FAD mice, and Avn-C contributes to mediating central immune homeostasis in obese 5×FAD mice, thereby suppressing the neuroinflammatory response.

### 3.6. Avn-C Modulates Inflammation-Related Gene Expression and Downregulates NOD1 Signaling in the Hippocampus

**Figure 7:**
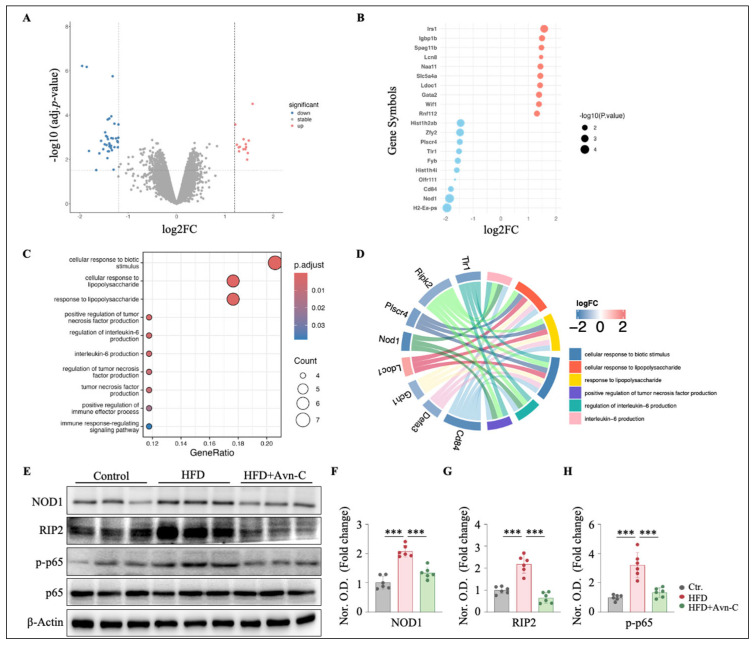
Avn-C treatment inhibits the NOD1/RIP2/NF-κB signaling pathway in the hippocampi of obese 5×FAD mice. (**A**) Differential expression analysis was performed on RNA-seq data. Volcano plot shows the DEGs (|FC| > 1.2, adj. *p*-value < 0.05) in the hippocampus (n = 3 per group from three animals). (**B**) Bubble chart shows the expression profiles of the top 20 DEGs in the hippocampus. (**C**) GO enrichment analysis was performed using R on 46 DEGs in HFD + Avn-C versus HFD. The most significantly represented GO term was ordered by adj. *p*-value < 0.05, and the activation of biological processes was assessed by calculating the GeneRatio (activated genes/total genes). (**D**) Circos plot shows eight genes’ expression, associated with at least three functional categories. Related genes were explored for their involvement in the top six functional categories. (**E**) Representative immunoblots showing the protein levels of NOD1, RIP2, p-p65, and p65 in the hippocampus. (**F**–**H**) Densitometry analysis and quantification of Western blots. One-way ANOVA: F (2, 15) = 41.9, *p* < 0.0001, η^2^ = 0.85; F (2, 15) = 36.7, *p* < 0.0001, η^2^ = 0.83; F (2, 15) = 29.1, *p* < 0.0001, η^2^ = 0.80. Data are expressed as means ± S.E.M., and statistical analysis was performed via Tukey’s post hoc test, *** *p* < 0.001.

To validate the underlying molecules and potential mechanisms of Avn-C in obesity-accelerated AD, a differential expression analysis was performed on RNA-seq data to determine the transcriptomic changes induced by Avn-C treatment. We identified 46 DEGs (defined as genes with |FC| > 1.2 and an adj. *p*-value < 0.05) in hippocampi from Avn-C-treated and untreated obese 5×FAD mice (Figure 7A), representing an alteration in the genome (46 genes in total ∼21,161 genes), including the top 10 downregulated genes (H2-Ea-ps, Nod1, Cd84, etc.) and 10 upregulated genes (Irs1, Igbp1b, Spag11b, etc.) (Figure 7B). Furthermore, the functional implications of these genes were explored using gene ontology (GO) enrichment analysis. A total of 102 GO terms were significantly overrepresented (*p* < 0.05) by the 46 genes, among which the largest functional cluster was associated with immune and inflammatory responses. This functional cluster consisted of a variety of biological processes, including the regulation of interleukin-6 production, tumor necrosis factor production, and the response to lipopolysaccharide and biotic stimuli, all of which were predicted to be strongly downregulated (*p* < 0.05) in Avn-C-treated obese 5×FAD mice (Figure 7C). We then annotated the relationships between specific genes and biological processes in the functional cluster. Gene–pathway interaction analysis enabled a comprehensive understanding of how Avn-C modulates HFD-induced neuroinflammation at the molecular level. Some key genes regulated in Avn-C treatment, including Nod1, Cd84, Tlr1, Plscr4, Ripk2, Plscr4, Defa3, Gch1, and Lodoc1, were clustered in at least three immune and inflammatory functions, such as the response to lipopolysaccharide, the regulation of tumor necrosis factor production, and interleukin-6 production (Figure 7D). Taken together, the transcriptomic analysis results suggest that Avn-C exerted a strong anti-inflammatory effect, which led to the downregulation of transcriptomic pathways, with implications for the immune and inflammatory responses. To validate the transcriptomic findings, Western blot analysis was conducted to assess the protein expression of key NOD1 pathway mediators. As shown in the quantified Western blots in Figure 7E, obese 5×FAD mice exhibited significantly increased expression of NOD1, RIP2, and phosphorylated NF-κB (p-p65) (Figure 7E–H) (NOD1, *p* < 0.001 vs. the control; RIP2, *p* < 0.001 vs. the control; and p-p65, *p* < 0.001 vs. the control). Avn-C treatment significantly reduced the expression of these proteins in obese 5×FAD mice (Figure 7F–H) (NOD1, *p* < 0.001 vs. HFD; RIP2, *p* < 0.001 vs. HFD; and p-p65, *p* < 0.001 vs. HFD), indicating that Avn-C effectively suppressed the activation of the NOD1/RIP2 signaling pathway.

### 3.7. NOD1 Activation Abolishes Avn-C-Mediated Memory Improvements in Obese 5×FAD Mice

**Figure 8:**
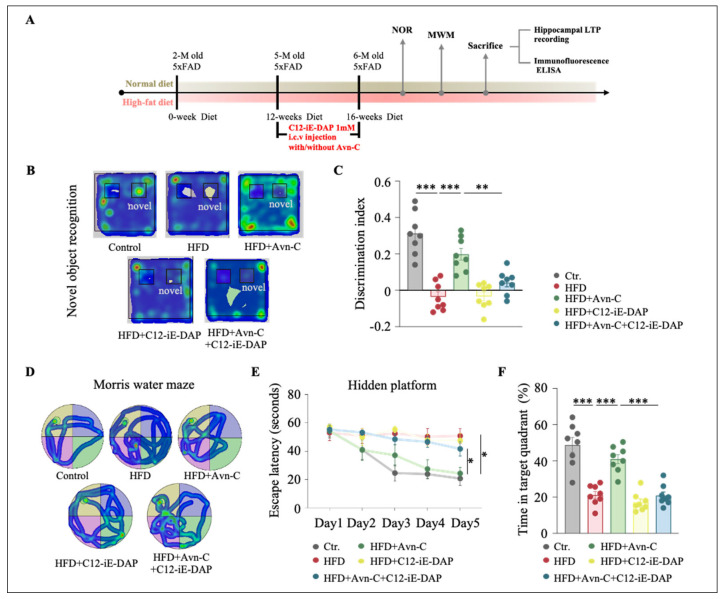
NOD1 activation abolishes Avn-C-mediated memory improvements in obese 5×FAD mice. (**A**) Graphical representation of experimental design. (**B**) Heatmap plots show the average cumulative time that 5×FAD mice spent in different parts in the NOR session. Red indicates longer times. Blue indicates shorter times. (**C**) Discrimination index expressed in seconds (n = 8 per group from eight animals). One-way ANOVA: F (4, 35) = 25.0, *p* < 0.0001, η^2^ = 0.74. (**D**) Heatmap plots show the escape strategy during the hidden platform task. Red indicates longer times. Blue indicates shorter times. (**E**) Escape latency and (**F**) time in the target quadrant (%) (n = 8 per group from eight animals). One-way ANOVA: F (4, 35) = 10.5, *p* = 0.01, η^2^ = 0.55; F (4, 35) = 29.1, *p* < 0.0001, η^2^ = 0.77. Data are expressed as means ± S.E.M., and statistical analysis was performed via Tukey’s post hoc test, * *p* < 0.05, ** *p* < 0.01, *** *p* < 0.001.

To determine whether the cognitive benefits of Avn-C in obese 5×FAD mice are mediated via NOD1 signaling, we examined the effects of C12-iE-DAP, a selective NOD1 agonist, on Avn-C-treated animals. As illustrated in the experimental timeline (Figure 8A), C12-iE-DAP (1 mM) was administered via i.c.v. injection during a 4-week Avn-C treatment period. Recognition memory was evaluated using the NOR test. As shown in the representative heatmaps (Figure 8B), Avn-C-treated obese 5×FAD mice spent more time exploring the novel object compared with normal diet-fed mice, whereas co-treatment with C12-iE-DAP reduced the novel object preference. Quantitative analysis revealed that the discrimination index was significantly lower in co-treated obese 5×FAD mice (*p* < 0.01, HFD + Avn-C + C12-iE-DAP vs. HFD + Avn-C) (Figure 8C), indicating that NOD1 activation blocked the beneficial effect of Avn-C on recognition memory. Spatial learning and memory were assessed using the MWM test. Representative swimming trajectories during the probe trial are shown in Figure 8D. Training performance revealed that Avn-C-treated mice exhibited shortened escape latencies compared with obese 5×FAD mice, while C12-iE-DAP co-treatment significantly delayed acquisition (*p* < 0.01, Figure 8E). In the probe test, Avn-C-treated mice displayed increased time spent in the target quadrant, which was significantly reversed by C12-iE-DAP (*p* < 0.001, Figure 8F). These findings suggest that the activation of NOD1 signaling by C12-iE-DAP negates the cognitive improvements conferred by Avn-C, highlighting the crucial role of the NOD1 pathway in mediating Avn-C’s beneficial effects.

### 3.8. Activation of NOD1 Attenuates the Therapeutic Efficacy of Avn-C in HFD-Fed 5×FAD Mice

**Figure 9:**
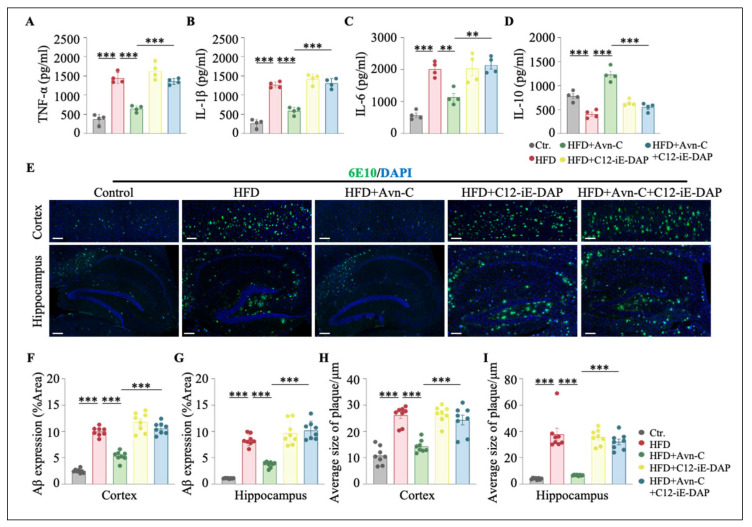
NOD1 activation inhibits the effects of Avn-C in reducing cytokine levels and Aβ deposition in obese 5×FAD mice. (**A**–**D**) ELISA results show the levels of TNF-α, IL-1β, IL-6, and IL-10 in the hippocampus (n = 4 per group from four animals). One-way ANOVA: F (4, 15) = 63.0, *p* < 0.0001, η^2^ = 0.94; F (4, 15) = 85.7, *p* < 0.0001, η^2^ = 0.96; F (4, 15) = 23.8, *p* < 0.0001, η^2^ = 0.86; F (4, 15) = 38.2, *p* < 0.0001, η^2^ = 0.91. (**E**) Representative images of Aβ plaque immunofluorescence staining in the cortex and hippocampus from each group. (**F**,**G**) Quantification of Aβ deposition (% Area) based on immunofluorescence staining in the cortex and hippocampus (n = 8 per group from four animals, two sections from each mouse). One-way ANOVA: F (4, 35) = 100.6, *p* < 0.0001, η^2^ = 0.92; F (4, 35) = 67.7, *p* < 0.0001, η^2^ = 0.89. Scale bar = 200 μm. (**H**,**I**) Average size of Aβ plaque (μm) was calculated based on immunofluorescence staining in the cortex and hippocampus (n = 8 per group from four animals, two sections from each mouse). One-way ANOVA: F (4, 35) = 32.0, *p* < 0.0001, η^2^ = 0.79; F (4, 35) = 45.7, *p* < 0.0001, η^2^ = 0.84. Scale bar = 200 μm. Data are expressed as means ± S.E.M., and statistical analysis was performed via Tukey’s post hoc test, ** *p* < 0.01, *** *p* < 0.001.

To determine whether the neuroprotective effects of Avn-C are mediated through the suppression of NOD1 signaling, we examined whether the pharmacological activation of NOD1 would compromise the anti-inflammatory effects and anti-Aβ burden of Avn-C in HFD-fed 5×FAD mice. The administration of C12-iE-DAP significantly elevated the hippocampal NOD1 protein levels; moreover, co-treatment with C12-iE-DAP attenuated the Avn-C-induced downregulation of NOD1, RIP2, and p-p65 (Figure S1). ELISA analysis further revealed that the reductions in pro-inflammatory cytokines (TNF-α, IL-1β, and IL-6) induced by Avn-C were significantly reversed by C12-iE-DAP co-administration (TNF-α, *p* < 0.001 vs. HFD + Avn-C; IL-1β, *p* < 0.001 vs. HFD + Avn-C; and IL-6, *p* < 0.01 vs. HFD + Avn-C) (Figure 9A–C). Although the IL-10 levels showed an increase after Avn-C treatment, this increase occurred upon NOD1 activation (*p* < 0.001 vs. HFD + Avn-C) (Figure 9D). It has been reported that inflammation is not only a result of AD but also a crucial player in this process [36]. Next, we examined whether the attenuation of Avn-C’s anti-inflammatory effect would be accompanied by a rebound in Aβ deposition. Here, 6E10 immunostaining showed that C12-iE-DAP treatment abolished the Avn-C-mediated reduction in the Aβ plaque burden in both the cortex and hippocampus (*p* < 0.001 vs. HFD + Avn-C) (Figure 9E–I). Overall, these findings suggest that the therapeutic effect of Avn-C in mitigating HFD-aggravated AD-related pathologies is driven by its anti-neuroinflammatory properties and may involve the NOD1/RIP2/NF-κB signaling pathway.

## 4. Discussion

Plant-based extracts are capable of countering cognitive decline via reductions in the expression of peripheral and neural inflammatory responses, blood–brain barrier leakage, and the activation of microglia and astrocytes [37]. In the present study, we demonstrate the neuroprotective effects of Avn-C treatment in HFD-fed 5×FAD male mice, which is a well-established model commonly used to evaluate the therapeutic potential of AD candidate agents [38,39]. In most AD model mice, females are more susceptible to plaques and tangles than male mice [40,41]; therefore, our choice of male mice was aimed at ensuring consistency and reducing confounding variables. In particular, this served to avoid the effects of estrogen on LTP levels and behavioral performance [42,43,44]. Our results demonstrate that HFD-induced obesity and insulin resistance exacerbated neuroinflammation, Aβ deposition, gliosis, synaptic dysfunction, and cognitive impairment in 5×FAD mice. Notably, our major finding is that four weeks of Avn-C treatment significantly inhibited neuroinflammation and attenuated HFD-aggravated AD pathological features. Moreover, Avn-C upregulated the expression of synapse-associated proteins such as Shank3, PSD95, and subunits of the AMPA and NMDA receptors. In addition, Avn-C treatment increased the dendritic spine density, promoted synaptic plasticity, and improved both recognition and spatial memory performance. Importantly, the ability of NOD1 activation to abolish the therapeutic effects of Avn-C suggests that the inhibition of the NOD1/RIP2 signaling pathway is a key mechanism underlying its neuroprotective effects. Based on these results, we speculate that Avn-C has a protective effect on HFD-accelerated AD.

HFD-induced obesity is widely recognized as a state of low-grade chronic inflammation that contributes to cognitive impairment and accelerates AD pathology [45,46]; however, therapeutic strategies targeting obesity-associated AD remain limited. Conventional anti-inflammatory agents such as sulindac and ibuprofen have been investigated in AD, but their application is constrained by long-term side effects [47,48]. Experimental evidence suggests that polyphenolic compounds, including flavonoids and phenylpropanoids, have anti-inflammatory properties [49,50]. In the present study, we observed that Avn-C attenuated HFD-aggravated neuroinflammation in obese 5×FAD mice, which was associated with improved cognitive performance. Consistent with prior reports showing that Avn-C suppresses NF-κB signaling and pro-inflammatory markers in AD models [33], our data further demonstrate that Avn-C significantly downregulated NOD1, RIP2, and p-p65 levels in the hippocampus. Moreover, the co-administration of a selective NOD1 agonist attenuated the anti-inflammatory effects of Avn-C, suggesting that Avn-C may exert its protective role, at least in part, through the modulation of NOD1/RIP2/NF-κB signaling in the context of HFD-induced AD pathology. Furthermore, an independent study showed that eight weeks of Avn-C supplementation attenuated exercise-induced inflammation in women following downhill running [27], suggesting the translational potential of Avn-C as a dietary intervention to modulate inflammation-related pathways.

NOD-like receptors, particularly NOD1 and NOD2, play a pivotal role in innate immunity and the regulation of inflammation. RIP2, a critical adaptor protein, mediates the responses of both NOD1 and NOD2 by triggering a shared pro-inflammatory pathway involving the activation of NF-κB and mitogen-activated protein kinase (MAPK) signaling cascades [51,52]. In humans, studies have demonstrated markedly increased expression of NOD1 in individuals with metabolic syndrome [53]. Consistent with these findings, feeding mice an HFD activates NOD1 signaling and triggers a pro-inflammatory response [54,55]. Our transcriptional analysis investigated the DEGs in Avn-C-treated and untreated obese 5×FAD mouse hippocampi, including Nod1, Ripk2, Tlr1, Ccrl2, and Cd84. Mediators of immune responses, such as Cd84, Defa3, Plscr4, Gch1, Zfp748, and Zfp97, were downregulated in Avn-C-treated obese 5×FAD mice. The Circos plot enabled the integrative visualization of the relationships between the DEGs and enriched biological processes, offering valuable insights into the mechanisms by which Avn-C exerts its protective effects against HFD-induced neuroinflammation. Notably, Nod1 and Ripk2 were associated with processes such as the regulation of interleukin-6 production, tumor necrosis factor production, and the response to lipopolysaccharide. Although our data suggest that the inhibition of the NOD1 signaling pathway is a key mechanism of Avn-C’s action, we cannot rule out the possibility that additional pathways, such as the modulation of the gut microbiota composition, peripheral immune responses, or systemic metabolic regulation, may also contribute to the observed effects. These potential mechanisms warrant further investigation.

Insulin signaling in the central nervous system is essential for cognitive functions such as learning and memory. Increasing evidence suggests that neuroinflammation may contribute to insulin resistance in the brain, thereby linking metabolic disorders to neurodegeneration [56,57,58]. Elevated levels of pro-inflammatory cytokines in both metabolic and neurodegenerative conditions may disrupt insulin signaling in the hippocampus [59]. While further investigation is required to determine whether CNS insulin resistance, alone or in combination with peripheral insulin resistance, contributes to cognitive dysfunction, experimental findings support the notion that enhancing hippocampal insulin signaling can mitigate memory impairments in both type II diabetes and AD [60]. In the present study, Avn-C treatment reduced the hippocampal levels of pro-inflammatory cytokines and increased the local insulin concentration in obese 5×FAD mice. Transcriptional analysis further revealed the upregulation of Irs1, consistent with the Western blot results showing the enhanced phosphorylation of IRS-1, Akt, and GSK-3β following Avn-C treatment. These findings suggest that Avn-C may improve hippocampal insulin sensitivity through anti-inflammatory mechanisms. Given that NOD1 activation is known to impair insulin signaling via the reduced phosphorylation of IRS-1 and downstream targets such as Akt and GSK-3β [61,62], our results support the possibility that NOD1 inhibition contributes to the insulin-sensitizing effects of Avn-C.

The accumulation of misfolded Aβ and hyperphosphorylated tau, along with glial activation, is a hallmark of AD pathology [63,64]. While previous studies reported that HFD did not significantly affect the Aβ burden in the hippocampus [65], our data show increased Aβ deposition in both the hippocampus and cortex in HFD-fed 5×FAD mice. This may be associated with impaired brain energy metabolism due to systemic insulin resistance, which has been implicated in AD pathogenesis [56,66]. Consistent with earlier studies [67], our results suggest that HFD-induced systemic metabolic dysfunction may contribute to synaptic deficits and exacerbate AD-related pathology. Additionally, we did not expect 5×FAD mice to develop neurofibrillary tangles because they did not display abnormally phosphorylated tau epitopes [68].

Dendritic spines are critical for synaptic signal integration and plasticity, and their loss is closely associated with cognitive decline in AD [69,70,71,72]. In HFD-fed AD models, synaptic protein levels are reduced, and the spine density is diminished, contributing to impaired memory performance [18,39,73,74]. In the present study, Avn-C treatment restored the expression of key postsynaptic proteins, including Shank3, PSD-95, and subunits of AMPA and NMDA receptors (GluA1, GluN1, and GluN2B). These molecular changes were accompanied by an increased dendritic spine density and improved performance in both object recognition and spatial memory tasks. Importantly, the co-administration of the NOD1 agonist C12-iE-DAP reversed the beneficial effects of Avn-C, suggesting that its synaptic and cognitive improvements are at least partially mediated through the inhibition of NOD1 signaling.

## 5. Conclusions

This study provides preclinical evidence that Avn-C may mitigate HFD-exacerbated AD-like pathology by reducing neuroinflammation and improving synaptic function. These effects appear to be mediated, at least in part, by the inhibition of NOD1/RIP2 signaling in the hippocampus. These findings highlight the need for further research to explore its mechanisms of action beyond NOD1 signaling, including its impacts on metabolic regulation and gut–brain interactions. Future research should evaluate the long-term safety, efficacy, and mechanistic pathways of Avn-C in both sexes and diverse experimental systems. While our results offer preliminary insights into the neuroprotective properties of Avn-C, its clinical relevance remains to be established.

## Data Availability

Data will be made available on request.

## Supplementary Materials

The following supporting information can be downloaded at https://www.mdpi.com/article/10.3390/nu17162679/s1. Figure S1: Activation of NOD1 signaling by C12-iE-DAP and its role in Avn-C–mediated NOD1/RIP2 pathway in the hippocampus of HFD-fed 5×FAD mice. (A,B) NOD1 protein expression increased after i.c.v. injection of C12-iE-DAP. (C–F) NOD1, RIP2, and p-p65 were upregulated in the hippocampus co-treated with Avn-C and C12-iE-DAP.
