# Supplementary material for: Avenanthramide-C Mitigates High-Fat Diet-Accelerated Alzheimer’s Pathologies via NOD1-Driven Neuroinflammation in 5×FAD Mice
Source: Nutrients. 2025 Aug 19;17(16):2679. doi: 10.3390/nu17162679 (PMC12388977; doi:10.3390/nu17162679)
Supplement: Supplementary file 1 [file nutrients-17-02679-s001.zip › nutrients-3780914-supplementary.pdf]

Supplementary figure

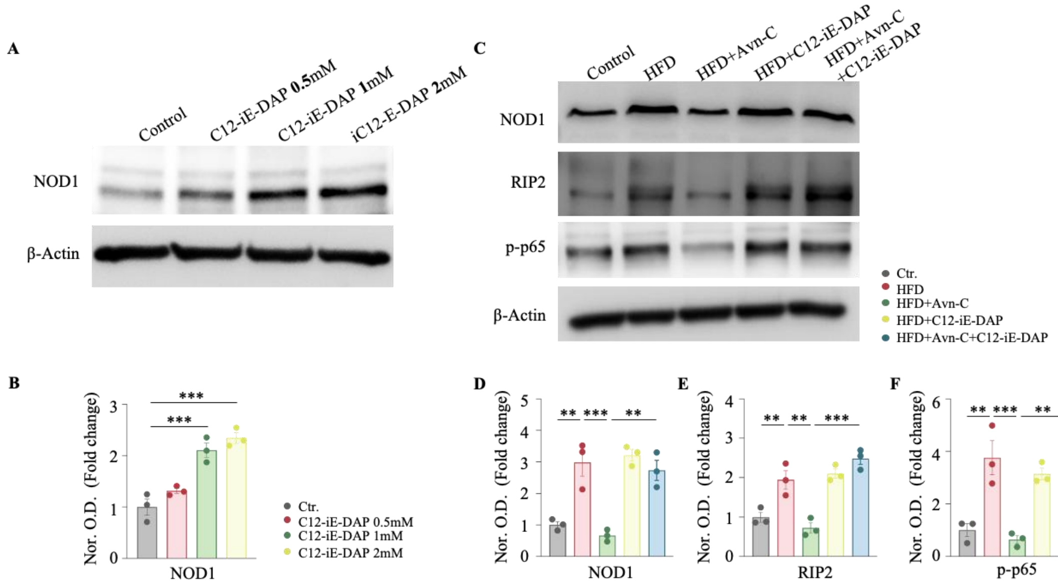

Figure S1. Activation of NOD1 signaling by C12-iE-DAP and its involvement in Avn-C-mediated NOD1/RIP2 signaling pathway in hippocampus from HFD-fed 5xFAD mice. (A) Representative immunoblots showing the NOD1 protein levels in the hippocampus after i.c.v injected with 0.5 mM, 1 mM, or 2 mM C12-iE-DAP. (B) Densitometry analysis and quantification of western blots (n = 3 per group from three animals). One-way ANOVA: F (3, 8) = 28.4, p = 0.0001. (C) Representative immunoblots showing the protein levels of NOD1, RIP2, and p-p65 in the hippocampus (D) to (F) Densitometry analysis and quantification of western blots (n = 3 per group from three animals). One-way ANOVA: F (4, 10) = 20.6, $p < 0.0001$; F (4, 10) = 23.1, $p < 0.0001$; F (4, 10) = 15.6, p = 0.0003. Data are expressed as means ± S.E.M and statistical analysis was performed by Turkey post-test, **$p < 0.01$, ***$p < 0.001$.
